# Increasing adverse event reports coincide with declining Medicare billings for interspinous devices

**DOI:** 10.1016/j.xnsj.2026.100875

**Published:** 2026-03-10

**Authors:** Aidan P. McAnena, Taylor McClennen, Raj J. Gala

**Affiliations:** a UMass Chan Medical School, University of Massachusetts, 55 N Lake Ave., Worcester, MA 01655, USA; b Department of Orthopaedics & Rehabilitation, Yale School of Medicine, 47 College St, New Haven, CT 06510, USA

**Keywords:** Spine, Interspinous spacers, Interspinous plates, Adverse events, Medical device, Database, Orthopedic surgery

## Abstract

**Background:**

ISDs treat spinal stenosis via a minimally invasive approach, but limited studies assess their efficacy and durability. The objective of this study was to characterize the incidence of adverse event reports (AERs) compared to Medicare claims for interspinous devices (ISDs).

**Methods:**

AERs for ISDs between 2017 and 2022 were downloaded from the Manufacturer and User Facility Device Experience (MAUDE) database. Reports were tabulated by brand, device problem, and patient problem. Centers for Medicare and Medicaid Services (CMS) files were surveyed to obtain the total number of Medicare procedures reported for the implantation of an ISD (Current Procedural Technology codes 22867-22870), serving as a proxy for the number of annual procedures.

**Results:**

A total of 1,009 MAUDE reports and 82,255 Medicare procedures were identified. Since 2019, ISD implantation procedures decreased by 47%, and the ratio of AERs to total procedures increased by 290%. X-Stop devices constituted the majority of AERs before 2020, but Superion had the majority after 2020. Reports from 2017 to 2020 frequently cited patient symptoms (>80%), whereas < 50% were symptomatic in 2021–2022. Before 2021, device problems were nonspecific, while from 2021 to 22, >40% of reports listed “material integrity problem.” Superion devices more often had material integrity and migration problems, while pain and vertebral fractures occurred across brands.

**Conclusions:**

The rate of AERs per ISD implantation procedure has increased since 2019. These findings suggest potential technological shortcomings, and ISDs may not be clinically viable for use in spinal surgery in their current form.

## Introduction

Spinal stenosis is a degenerative condition that leads to narrowing of the spinal canal. Studies have demonstrated that approximately 103 million people worldwide, and 11% of older adults in the US, are affected by lumbar spinal stenosis [1]. With the aging population, the prevalence of spinal stenosis is predicted to increase to almost 18 million in the next decade. Among adults above 65 years who are undergoing spine surgery, lumbar spinal stenosis is the leading diagnosis [2].

Interspinous spacers and plates treat spinal stenosis via a minimally invasive approach. These devices are placed between adjacent spinous processes to produce relative local kyphosis, stretching the ligamentum flavum, and hopefully lead to a greater canal and foramen diameter. Their goal is to improve the symptoms of neurogenic claudication, potentially bridging the gap from epidural injection to lumbar laminectomy. Different spacers may have subtle different indications, but broadly they are used in the setting of lumbar stenosis, one or sometimes two levels, and some of them can be used even with stable spondylolisthesis [3].

Evidence demonstrates mixed outcomes regarding the efficacy and long-term durability of interspinous devices (ISDs). Multiple studies have demonstrated higher reoperation rates for ISDs compared to decompression surgery [4,5]. Similarly, the American Society of Pain and Neuroscience guidelines highlight that while ISDs can be effective, they are also associated with higher complication and reoperation rates when compared to decompression surgery [6]. Given the mixed outcomes in the literature with regards to the risk versus benefit analysis for ISDs, it is crucial to further elaborate the evidence that supports or contradicts these devices.

One opportunity for investigating adverse outcomes from ISDs is the Manufacturer and User Facility Device Experience (MAUDE) database. The MAUDE database is a public collection of medical device reports from the United States Food and Drug Administration (FDA). The MAUDE database contains adverse event reports (AER) from both mandatory (manufacturers, importers, device use facilities) and voluntary (health care professionals, patients, consumers) reporters. Each AER includes the date of event, patient problem, device problem, brand name of the device, and narrative description of the event [7].

In addition to the MAUDE database, other databases may aid in understanding the usage of ISDs. One such database is the Part B National Summary Data File, an annual data file published by the Centers for Medicare and Medicaid Services (CMS). This data file reports the number of Current Procedural Technology (CPT) codes documented to process insurance claims [8].

It is critical for physicians and patients to be well-informed on the risks and benefits of ISDs. Although some studies have reported on the frequency of AERs over various time frames, none of these previous works included data about the total number of procedures per year, thus limiting the conclusions that can be drawn [[9], [10], [11]]. The MAUDE database and the CMS data files provide a wealth of knowledge regarding the overall usage and AERs associated with ISD placement. Therefore, the objective of this study was to investigate trends in AERs for ISDs compared to their annual usage from 2017 to 2022.

## Methods

**Fig. 1:**
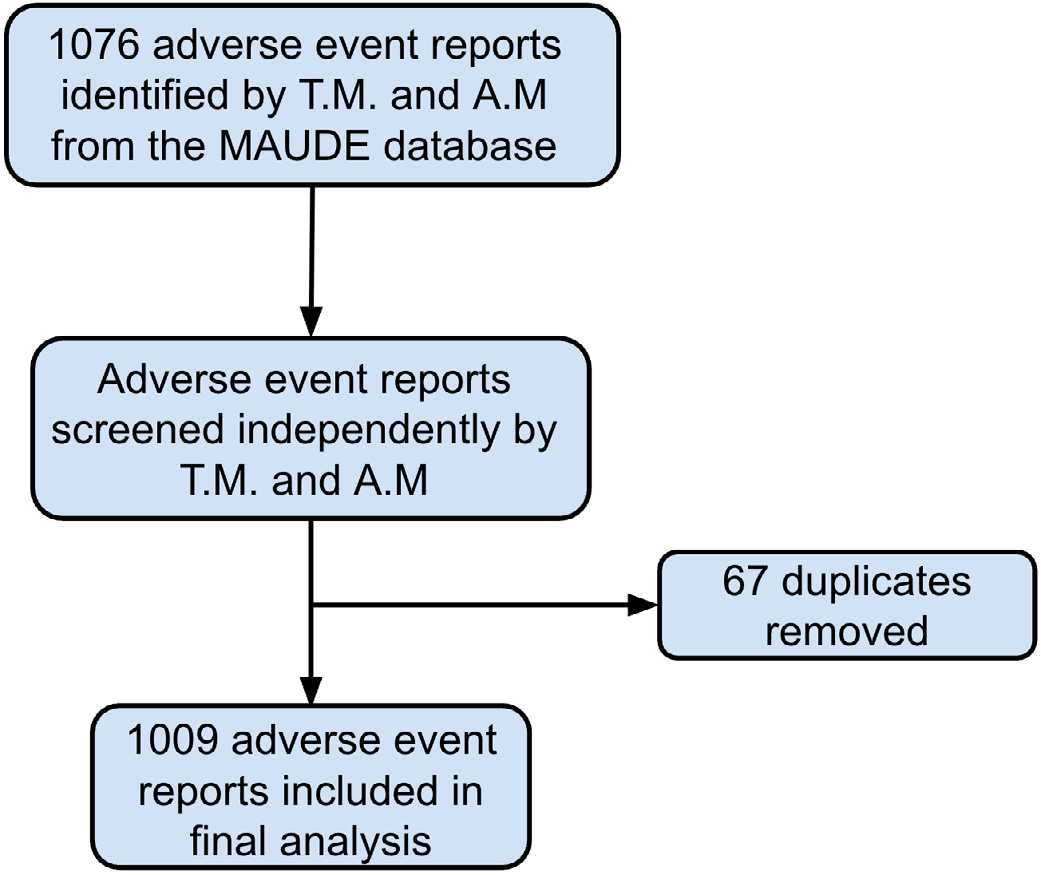
Adverse event reports flowchart. Adverse event reports from the Manufacturer and User Facility Device Experience (MAUDE) database for the device “Prosthesis, Spinal Process Spacer/Plate” between January 1, 2017 and December 31, 2022 were downloaded. Reports were independently screened by two reviewers, and duplicates were removed. A total of 1,009 adverse event reports were included in the final analysis.

The FDA MAUDE database was queried for reports associated with the product class “Prosthesis, Spinal Process Spacer/Plate” between January 1, 2017 and December 31, 2022. Reports were downloaded and tabulated according to device brand, device problem, patient problem, and event narrative. Event narrative and event date were used to identify and remove duplicate reports (Fig. 1).

The Part B National Summary Data File database from the CMS were surveyed from 2017 to 2022. The CPT codes 22867-22870 were used to obtain the total number of Medicare procedures reported for the implantation of an ISD. Since Medicare serves the appropriate demographic for spinal stenosis, this data was used as a proxy for the total number of procedures in a given year.

AERs were tabulated by year to identify temporal trends, as well as for each device brand. Data extracted from each AER included Report Number, Event Date, Event Type, Manufacturer, Brand Name, Device Problem, Patient Problem, and Event Text (narrative description of each event). Annual AER incidence was compared to the number of Medicare codes billed each year for the number of interspinous implants. Data collection and analysis was performed using Microsoft Excel version 16.94.

## Results

A total of 1,009 MAUDE adverse event reports and 82,255 Medicare procedures were identified through review of the MAUDE database and the CMS Part B National Summary database (Fig. 1). Each AER included the event date, event type, manufacturer and brand name, device problem, patient problem, and a narrative description of the event. The event type was categorized as either “Injury” or “Malfunction.” Device brand included three ISDs: Coflex (XTant Medical, Belgrade, Montana; FDA approval 10/2012), Superion (Boston Scientific, Marlborough, Massachusetts; FDA approval 5/2015), and X-Stop (Medtronic, Minneapolis, Minnesota; FDA approval 11/2005). Patient problems included “no clinical signs, symptoms or conditions,” “pain,” and “vertebral fracture,” as well as other problems specific to each AER. Finally, the device problem was listed as “material integrity problem,” “adverse event without identified device or use problem,” “lack of effect,” “migration,” “device dislodged,” or “other.” AERs were tabulated according to each of these characteristics.

**Fig. 2:**
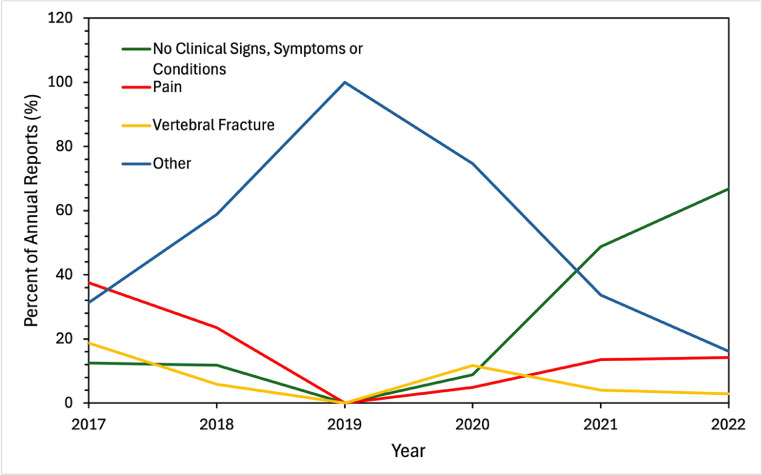
Patient problem for adverse event reports in the Manufacturer and User Facility Device Experience (MAUDE) database for interspinous devices. All data was collected annually for the years 2017–2022 and reported as percent of total reports. *N* = 1,009.

**Fig. 3:**
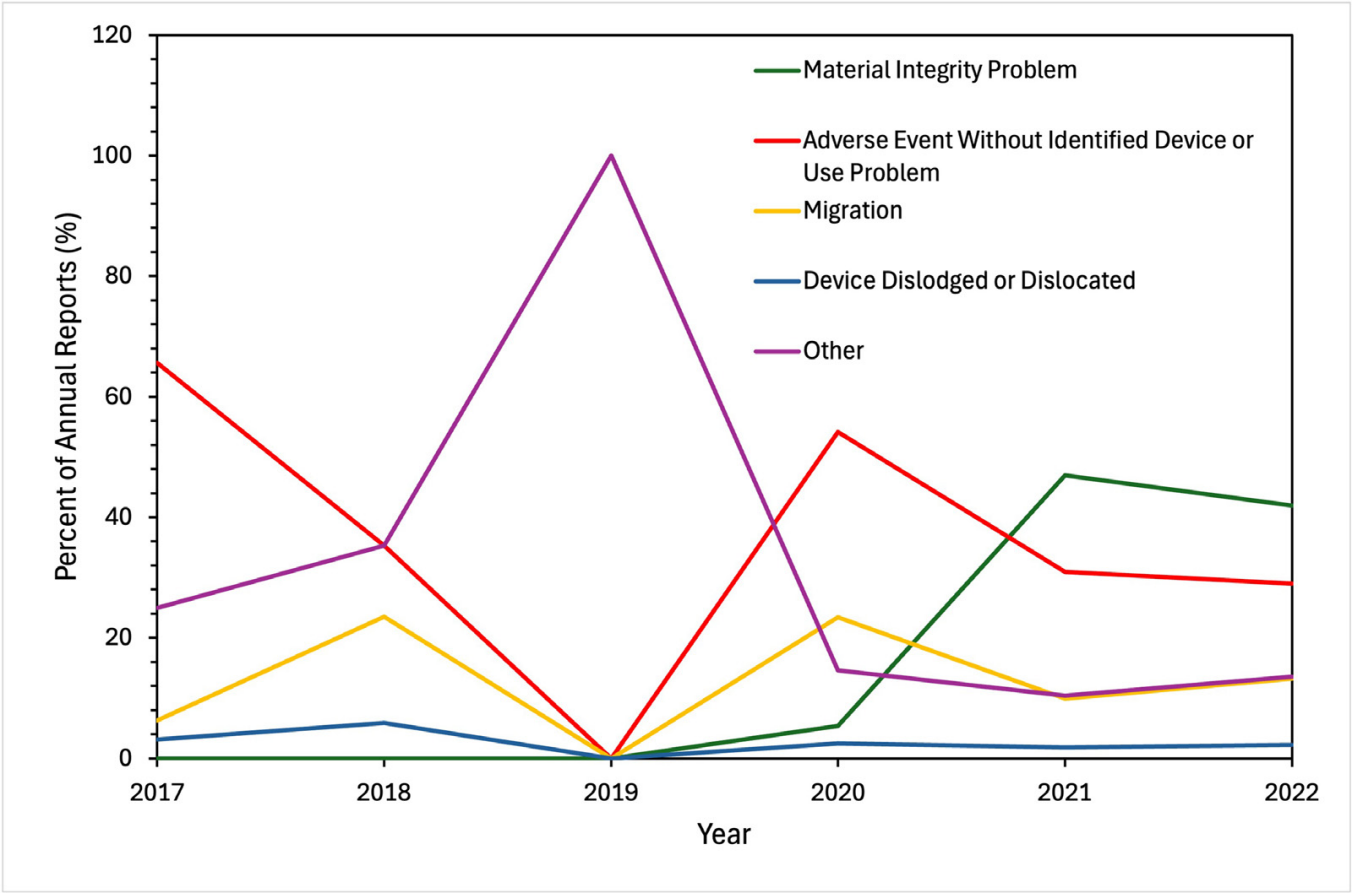
Device problem for adverse event reports in the Manufacturer and User Facility Device Experience (MAUDE) database for interspinous devices. All data was collected annually for the years 2017–2022 and reported as percent of total reports. *N* = 1,009.

Patient problems were most commonly reported as “no clinical signs or symptoms” (42.6%) or multiple issues (36.4%), but the most commonly listed singular problems were pain (17%) and vertebral fracture (4%). Earlier AERs (2017–2020) were more likely to report patient symptoms, with more than 85% of the AERs indicating a clinical symptom (rather than “no clinical signs, symptoms, or conditions”). In contrast, later reports (2021–22) were less likely to claim patient symptoms, with symptoms reported in less than 50% of AERs (Fig. 2). The most common device problems overall were “material integrity problem” (34.6%) and “adverse event without identified device or use problem” (36.2%). Migration was also common (13.8%). Before 2021, device problems were largely nonspecific, with “adverse event without identified device or use problem” or “other” as the leading inputs. However, from 2021 to 2022, over 40% of AERs listed the device problem as “material integrity problem” (Fig. 3). Overall, reports from 2021 to 2022 were more likely to cite asymptomatic patients and material integrity issues.

**Fig. 4:**
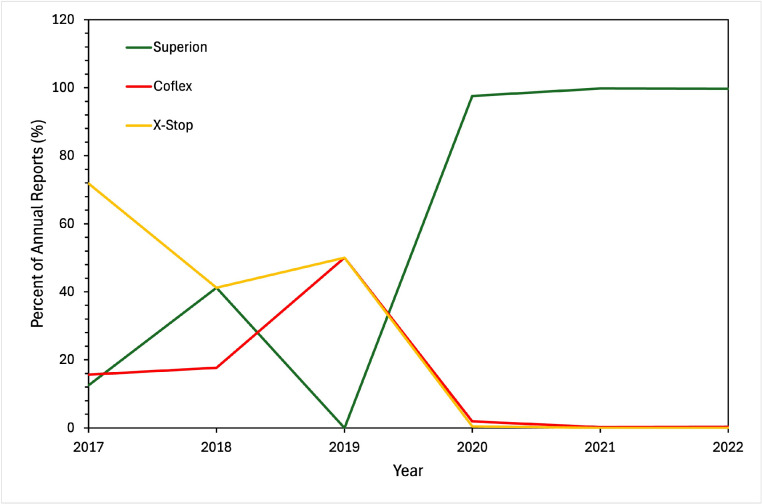
Device brand for adverse event reports in the Manufacturer and User Facility Device Experience (MAUDE) database for interspinous devices. All data was collected annually for the years 2017–2022 and reported as percent of total reports. *N* = 1,009. Interspinous devices includes Superion (Boston Scientific), Coflex (XTant Medical), and X-Stop (Medtronic).

With regards to device brand, Superion and X-Stop comprised the majority of reports. In 2019 and prior, the majority of the AERs each year were for X-Stop (41%–75%). After the removal of X-Stop from the market in 2015, the percentage of AERs arising from Superion devices increased from 12.5% in 2017 to 97.6% in 2022 (Fig. 4). Patient and device problems were also analyzed individually for each brand of ISD (Table 1, Table 2). For Coflex devices, most device problems were categorized as “other” which often meant insufficient information but sometimes meant device break or fracture. The most common issue for Superion devices was “material integrity problem” (36.5%), followed by “adverse event without identified device or use problem” (35.6%). Migration occurred in 14% of Superion reports but only six times for X-Stop (18.8%) and once for Coflex (6.7%). In terms of patient problems, many reports cited “no clinical signs or symptoms,” almost all of which came from Superion (*N* = 429, 44.6% of Superion reports). Both pain and vertebral fractures were prevalent for all three devices.

**Table 1:** Device problems reported in the MAUDE* database, stratified by interspinous device brand.

Device problem	Coflex	Superion	X-Stop
	*N* = 15	*N* = 962	*N* = 32
	N (%)		
Material integrity problem	0 (0)	351 (36.5)	0 (0)
Adverse event without identified device or use problem	1 (6.7)	342 (35.6)	23 (71.9)
Migration	1 (6.7)	135 (14)	6 (18.8)
Fracture	3 (20)	0 (0)	0 (0)
Device dislodged or dislocated	1 (6.7)	22 (2.3)	0 (0)
Other/multiple problems	9 (60)	112 (11.6)	3 (9.4)

⁎ Manufacturer and user facility device experience.

**Table 2:** Patient problems reported in the MAUDE* database, stratified by interspinous device brand.

Patient problem	Coflex	Superion	X-Stop
	*N* = 15	*N* = 962	*N* = 32
	N (%)		
No clinical signs, symptoms, or conditions	1 (6.7)	429 (44.6)	0 (0)
Pain	4 (26.7)	156 (16.2)	12 (37.5)
Vertebral fracture	3 (20)	35 (3.6)	2 (6.25)
Other/multiple problems	7 (46.7)	342 (35.6)	18 (56.3)

⁎ Manufacturer and user facility device experience.

**Fig. 5:**
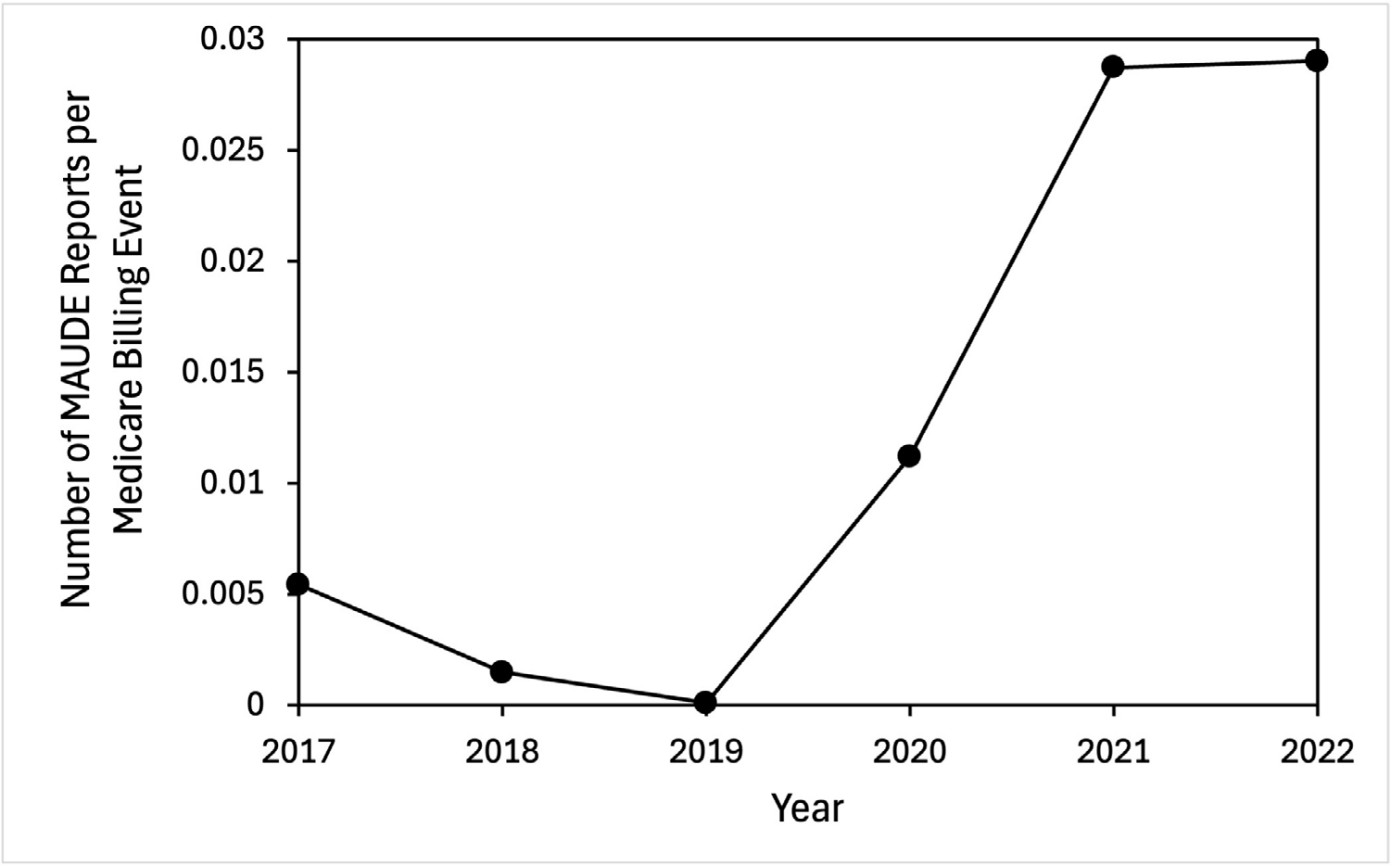
Annual Adverse event reports compared to Medicare-billed interspinous device placements from 2017 to 2022. Adverse event reports were downloaded from the Food and Drug Administration Manufacturer and User Facility Device Experience (MAUDE) database. Medicare billing data was obtained from the Centers for Medicare and Medicaid Services Part B National Summary Data File database, using the CPT codes 22867–22870. Adverse event reports are shown as a percentage of total Medicare billed-procedures per year.

The number of ISD procedures billed by Medicare increased to a peak in 2019, then began to fall. In 2017, only 6,274 procedures were billed, compared to 20,027 in 2019. In 2020 the total began to decline to 18,327, with a sharp decrease to 10,682 in 2022. Overall, the number of ISD procedures billed by Medicare has decreased 47% since 2019. In comparison, the number of AERs in MAUDE reached a peak in 2021. Initial reports were minimal with only 34 AERs in 2017, 17 in 2018, and 2 in 2019. However, there was a notable increase in 2020 with 205 reports, and the following year saw 443 reports in 2021. Following this increase the numbers began to decline, with 310 reports in 2022. Overall, Medicare procedures reports peaked in 2019, while MAUDE adverse reports were at maximum in 2021 (Fig. 5).

The number of AERs compared to the number of ISD procedures billed by Medicare has greatly increased in the past four years (Fig. 5). In 2019, the number of AERs was less than 1% of the Medicare billings. However the following years saw a rapid increase, with 2020 reporting 1.1%, and both 2021 and 2022 with AERs comprising 2.9% of billed Medicare procedures. Overall, from 2019 to 2022, there was a 290% increase in interspinous spacer AERs relative to Medicare billing.

## Discussion

Although ISDs have become a popular method of treating lumbar spinal stenosis, they require a critical analysis of their safety and efficacy. In this study we retrospectively analyzed 1,009 adverse event reports from the FDA MAUDE database. After 2020, patient problems were more commonly reported as asymptomatic or without clinical signs and symptoms. Furthermore, after 2020, the device problem was most often listed as “material integrity problem,” while prior years reported a wider spread of issues. The second most common device problem after 2020 was “adverse event without identified device or use problem.” According to the International Medical Device Regulators Forum, this description indicates that “an adverse event (e.g. patient harm) appears to have occurred, but there does not appear to have been a problem with the device or the way it was used [12].” Therefore this category represents negative patient outcomes that are unrelated to the device itself, and cannot be used to draw conclusions about device safety.

Regarding device brand, X-Stop comprised the majority of reports until its removal in 2015, whereas after 2020 the Superion system accounted for greater than 90% of reports. The trend in patient and device problems may be due to the increase in Superion reports, as Superion AERs were more often reportedly asymptomatic and had material integrity problems.

We also analyzed patient and device problems by brand. Material integrity problems were most frequent for Superion ISDs, and migration occurred almost exclusively in Superion device reports. Notably, lack of effect was never cited as the device problem for any brand. For patient problems, 44.6% of Superion reports cited “no clinical signs or symptoms.” There is not a clear trend indicating that one brand predisposes patients to pain or vertebral fracture. Superion devices have higher rates of migration and material integrity problems than Coflex and X-Stop devices, and these reports are largely in asymptomatic patients. Coflex and X-Stop are not without issue, however, as over 40% of reports for both endorse pain or vertebral fracture as the patient problem. Different brands predispose patients to different issues, but all ISDs have resulted in morbidity, according to available MAUDE data.

The increase in adverse event reports for ISDs raises questions about their safety in surgical use. These findings corroborate with other studies indicating that ISDs are more likely to require surgical revision. One study analyzed reports in the MAUDE database and demonstrated that there is a high likelihood of revision surgery related to complications that can be attributed specifically to the ISD [10]. One of these complications is fracture, which has been reported to have high incidence in the early postoperative period. Another prospective observational study found that 28.9% of patients experienced spinous process fractures after ISD implantation, with many asymptomatic and not detectable on plain radiographs [13]. This evidence supports the present study’s finding that AERs are increasingly presenting with no specific clinical signs or symptoms. Furthermore, studies analyzing multiple databases, including Medicare claims data and the MarketScan database, found that ISDs had higher rates of revision surgery when compared to decompression or fusion [[14], [15], [16]]. Overall, the literature supports the findings here that ISDs must be more rigorously examined given the high rate of associated adverse events.

### Limitations

This report is not without limitations. First, the MAUDE database only includes the data that is voluntarily reported, which may lead to inaccurate counts of AERs. This furthermore may lead to misrepresentation of the number of AERs for each device problem, patient problem, or brand. In addition, although AERs contain a narrative of the event, they do not include certain details such as the patient’s medical history, bone composition, anatomic abnormalities, or other information that may affect the individual outcome of the procedure. Most adverse event reports additionally came from X-Stop, which has been removed from the market and is no longer in use. The conclusions of this article should be interpreted in the context of the devices currently on the market.

The Medicare CMS database may also not represent the full quantity of ISDs that were placed in a given year, as some patients may have a private insurer, be younger than 65, or otherwise outside of the Medicare criteria. No studies directly quantify the proportion of interspinous device implantations procedures that are represented by the Medicare procedures. However, one article examines the distribution of spinal fusion usage among insurance payer groups in the United States from 1997 to 2014. This study found that 29.9% of total discharges with spinal fusion were covered by Medicare, while 50.3% were covered by private insurance, 6% by Medicaid, and 1.5% uninsured [17]. This percentage has likely increased since 2014 due to the aging population of the United States that are covered by Medicare. Given this data, it is reasonable to estimate that for interspinous device implantation, Medicare claims represent over one-third of the total number of annual procedures. Therefore, the present study potentially overestimates the annual ratio of AERs to ISD implantation procedures. However, this overestimation is consistent across each year included in the analysis, thus the conclusions drawn from the overall trend remain valid.

Finally, the nature of the MAUDE database does not allow determination of causality, incidence, or comparative risk. The reported increase in adverse event reports relative to Medicare claims may reflect several causes, including but not limited to: heightened awareness of device-related issues, increasing regulatory scrutiny, changes in adverse event reporting behavior, and postmarket surveillance bias. Additionally, the decrease in ISD utilization may relate to removal of devices from the market, as the number of Medicare billings has also decreased. A more granular dataset would be necessary to determine the clinical viability of ISDs.

## Conclusions

The rate of AERs for ISDs is increasing while the implementation of these devices is concurrently decreasing, with high prevalence of material integrity problems. Patient and device problems vary by brand, but complications were reported for all ISDs. Although some interspinous devices have been removed from the market, the increasing ratio of AERs to Medicare procedure billings suggest that caution should be exercised when implementing these devices. These findings corroborate previous literature indicating high rates of adverse events with ISDs, which may be inferior to traditional surgical options such as laminectomy. Overall, there is a growing amount of data indicating that interspinous spacers and plates may have a high rate of complications, with recent decreases in clinical implementation. The use of ISDs in spinal surgery should be selected only after a thorough consideration of risks and benefits in each individual case, and additional clinical studies are needed to clarify the safety and benefits of ISDs.

## Data Availability

The datasets generated during and/or analyzed during the current study are available in the Food and Drug Administration Manufacturer and User Facility Device Experience (MAUDE) (https://www.accessdata.fda.gov/scripts/cdrh/cfdocs/cfmaude/search.cfm), and the Centers for Medicare and Medicaid Services (CMS) Part B National Summary Data File (https://www.cms.gov/data-research/statistics-trends-and-reports/part-b-national-summary-data-file).

## Author contributions

Aidan McAnena: Conceptualization, methodology, formal analysis, writing - original draft. Taylor McClennen: Conceptualization, methodology, formal analysis, writing - original draft. Raj Gala: Conceptualization, writing - review and editing, supervision.

## Declaration of competing interest

The authors declare that they have no known competing financial interests or personal relationships that could have appeared to influence the work reported in this paper.
